# Biophysical characterization and modulation of Transthyretin Ala97Ser

**DOI:** 10.1002/acn3.50887

**Published:** 2019-09-10

**Authors:** Yo‐Tsen Liu, Yueh‐Jung Yen, Frans Ricardo, Yu Chang, Pei‐Hao Wu, Shing‐Jong Huang, Kon‐Ping Lin, Tsyr‐Yan Yu

**Affiliations:** ^1^ Division of Epilepsy Neurological Institute Taipei Veterans General Hospital Taipei Taiwan; ^2^ National Yang‐Ming University School of Medicine Taipei Taiwan; ^3^ Institute of Brain Science National Yang‐Ming University Taipei Taiwan; ^4^ Brain Research Center National Yang‐Ming University Taipei Taiwan; ^5^ Institute of Atomic and Molecular Sciences Academia Sinica Taipei Taiwan; ^6^ Instrumentation Center National Taiwan University Taipei Taiwan; ^7^ Division of Peripheral Nervous System Disorders Neurological Institute Taipei Veterans General Hospital Taipei Taiwan; ^8^ International Graduate Program of Molecular Science and Technology National Taiwan University Taipei Taiwan

## Abstract

**Objective:**

Ala97Ser (A97S) is the major transthyretin (*TTR)* mutation in Taiwanese patients of familial amyloid polyneuropathy (FAP), characterized by a late‐onset but rapidly deteriorated neuropathy. Tafamidis can restore the stability of some mutant TTR tetramers and slow down the progression of *TTR*‐FAP. However, there is little understanding of the biophysical features of A97S‐TTR mutant and the pharmacological modulation effect of tafamidis on it. This study aims to delineate the biophysical characteristics of A97S‐TTR and the pharmacological modulation effect of tafamidis on this mutant.

**Method:**

The stability of TTR tetramers was assessed by urea denaturation and differential scanning calorimetry. Isothermal titration calorimetry (ITC) was used to measure the binding constant of tafamidis to TTR. Nuclear magnetic resonance spectroscopy (NMR) titration experiment was used to map out the tafamidis binding site.

**Results:**

Chemical and thermal denaturation confirmed the destabilization effect of A97S. Consistent with other the amyloidogenic mutant, A97S‐TTR has slightly lower conformational stability. NMR revealed the binding site of A97S‐TTR with tafamidis is at the thyroxine binding pocket. The ITC experiments documented the high affinity of the binding which can effectively stabilize the A97S‐TTR tetramer.

**Interpretation:**

This study confirmed the structural modulation effect of tafamidis on A97S‐TTR and implied the potential therapeutic benefit of tafamidis for A97S *TTR*‐FAP. This approach can be applied to investigate the modulation effect of tafamidis on other rare *TTR* variants and help to make individualized choices of available treatments for FAP patients.

## Introduction

Familial amyloid polyneuropathy (FAP) is the most common hereditary amyloid diseases. Transthyretin‐related FAP (*TTR*‐FAP) is the most prevalent genetic subtype characterized by debilitating polyneuropathy and life‐threatening cardiomyopathy.^1^, ^2^ The disease‐causing gene is *TTR* (NM_000371). More than 150 disease‐causing *TTR* mutations associated with highly variable clinical manifestations have been identified.^2^ Val30Met (V30M) is the most frequent mutation worldwide.^2^, ^3^ V30M *TTR*‐FAP typically presents by a slowly progressive polyneuropathy with the onset at around 30 years and most patients could still walk independently even after 20 years of disease onset. Cardiac manifestations usually occur in the later stage of the disease.^4^, ^5^, ^6^, ^7^ The overall survival rate could stay around 80% after 10 years of follow‐up.^4^ It has been reported that Ala97Ser (A97S) is the major *TTR* mutation, accounting for 91.2% of the hereditary transthyretin amyloidosis (ATTR) pedigrees.^8^, ^9^, ^10^ Contrary to the classical early‐onset V30M *TTR*‐FAP, the patients with A97S *TTR*‐FAP have a much later onset with the mean age of onset of 58.2 years but would become bed ridden or wheel‐chair dependent within 8.4 years of the onset on average.^8^, ^9^, ^10^ The rapidly deteriorative course may be partially due to severe cardiac dysfunction. Life‐threatening cardiac arrhythmia (15.1%) and heart failure (16.4%) are prevalent in the A97S patients and might become severe before the full brown of peripheral neuropathy.^8^, ^10^ Although only a limited number of cases were available to provide a longitudinal follow‐up information, unfavorable events like syncope/cardiac arrest/ cardiopulmonary‐cerebral resuscitation tended to occur approximately 4 years after the appearance of the initial neurological symptoms and up to half died within 11 years after the first symptom or event.^9^

The encoding protein of *TTR* is transthyretin (TTR), a 55‐kDa homo‐tetrameric plasma protein circulating in the human serum and cerebrospinal fluid. The toxic species of most pathogenic *TTR* mutations is comprised of mutant monomers with a lower conformational stability and a higher propensity to dissociation from the tetramer then leading to amyloid fibril formation.^11^, ^12^ Tafamidis, one of the TTR stabilizers, has shown the potency to slow down the progression of neuropathy with a long‐term safety and to reduce the mortality and hospitalizations due to TTR amyloid cardiomyopathy (TAC).^13^, ^14^, ^15^, ^16^, ^17^, ^18^, ^19^, ^20^ However, only very limited patients of A97S *TTR*‐FAP were recruited in previous clinical trials because of the late‐onset nature and the moderate to severe disability at diagnosis. So far there is little information of the effect of tafamidis on A97S *TTR*‐FAP.

Understanding the biophysical characteristics of A97S‐TTR mutant and the modulation effects by tafamidis will help understand the pathogenicity of this mutation and the potential benefits from the pharmacotherapy. In this study, we assessed the stability of the A97S‐TTR tetramer by monitoring urea denaturation and measuring the melting temperature using differential scanning calorimetry (DSC). Isothermal titration calorimetry (ITC) was used to measure the binding constants of tafamidis to A97S‐TTR. Solution nuclear magnetic resonance (NMR) spectroscopy was used to map out the structure and the tafamidis binding site of A97S‐TTR. Our results will generate significant impacts on the clinical management and influence the healthy policy for patients of A97S *TTR*‐FAP. The approach can be applied to evaluate the potential benefits of pharmacological modulation for other *TTR* variants.

## Materials and Methods

### TTR unfolding assay using urea

Proteins unfolding of WT‐TTR, A97S‐TTR, T119M‐TTR, V30M‐TTR, and L55P‐TTR were assessed using urea denaturation assay.^21^ TTR sample solutions (0.1 mg/mL) were prepared by diluting 200 μL of 1.0 mg/mL TTR stock into 1.8 mL buffer, containing 10 mmol/L Na phosphate (pH7.5) with 100 mmol/L KCl, 1 mmol/L EDTA, 1 mmol/L DTT, 0.02 % sodium azide, and various concentrations of urea. The fluorescence emission ratio at 355 and 335 nm (F355/F335) of each sample was then monitored using a fluorescence spectrometer (Perkin Elmer LS‐55) after 96 h of incubation at room temperature. The excitation slits were set at 2.5 nm, while the emission slits were set at 3.5 nm. Excitation wavelength was fixed at 295 nm and the emission spectra were collected from 310 to 450 nm.

### Differential scanning calorimetry (DSC)

DSC measurements were performed on a Nano DSC differential scanning microcalorimeter (TA Instruments). By measuring the critical concentration for different TTR variants to re‐associate to be TTR tetramer, we can provide a parameter to assess the tendency of different TTR proteins to be in tetrameric state. In addition to WT‐TTR, A97S‐TTR, V30M‐TTR, and L55P‐TTR, the TTR protein with the protective variants R104H and T119M were also studies. Experiments were carried out at the concentrations of the TTR protein ranging from 1.5 to 2 mg/mL at the scan rate of 1.0°C/min. Protein samples were prepared in PBS buffer, as well as in PBS buffer containing 5% DMSO. To examine the effect of tafamidis‐binding on the *Tm* of TTR proteins, we needed to ensure that tafamidis can be completely solubilized. Thus, PBS buffer containing 5% DMSO was also used in the experiment. For DSC experiments of tafamidis bound TTR, the ratio of tafamidis to TTR tetramer equaled to 1.6 and only PBS buffer containing 5% DMSO was used. Before the measurements, the sample and reference solutions were properly degassed. An overpressure of 3 atm was always kept over the liquid in the cells throughout the scans to prevent any degassing during heating. The buffer scan (i.e., instrumental baseline) was determined before each sample scan, by filling both the sample and reference cells with the buffer used for the protein sample and using the same scanning parameters.

### Isothermal titration calorimetry (ITC)

ITC experiments were performed for WT‐TTR, the TTR mutants of A97S, V30M, and L55P based on Bulawa et al 2012.^14^ The detailed experimental conditions were documented in Table S1. Dissociation constants for tafamidis and each TTR protein were determined using a Microcal VP‐ITC isothermal titration calorimeter (Microcal Inc.). The concentrations of proteins and tafamidis used in all ITC experiments are listed in Table S1. The initial injection of 2.5 µL of tafamidis solution was followed by 49 injections of 5 µL each (25°C). The integrated heat was plotted against the molar ratio of tafamidis added to protein in the cell, yielding complete binding isotherms.

### Nuclear magnetic resonance spectroscopy

NMR spectroscopic characterization of the structural changes by tafamidis binding to A97S‐TTR and WT‐TTR was performed. NMR experiments were carried out at 37°C on Bruker spectrometers, operating at 600 MHz, 800 MHz and 850 MHz (proton frequencies.) Each NMR spectrometer was equipped with a Bruker TXI probes. Transverse relaxation‐optimized spectroscopy (TROSY) based triple‐resonance NMR experiments, including TROSY‐HNCA, TROSY‐HN(co)CA, TROSY‐HNCACB and NOESY‐TROSY‐HSQC were recorded for sequence‐specific resonance assignment.^22^ A typical NMR sample for this purpose contained 0.6–0.9 mmol/L TTR in a buffer containing 25 mmol/L Na phosphate, 100 mmol/L NaCl, 0.5 mmol/L EDTA, 1 mmol/L TCEP at pH 6.5. The TROSY‐based ^1^H‐^15^N heteronuclear single quantum coherence spectroscopy (TROSY‐HSQC) spectra of TTR with and without the addition of tafamidis were recorded to analyze the chemical shift changes due to drug binding. To reliably assign the peaks shift due to the binding with tafamidis, TROSY‐HNCA spectra of both samples were also recorded. The NMR sample buffer for this purpose contained an additional 5% of DMSO in order to completely dissolve tafamidis at 2.5:1 molar ratio (tafamidis to TTR) to saturate the tafamidis binding sites of TTR. The NMR data were processed with NMRPipe and the assignments were made using the program CARA.^23^

## Results

### Destabilization effect of A97S‐TTR mutant in chemical denaturation

**Figure 1:**
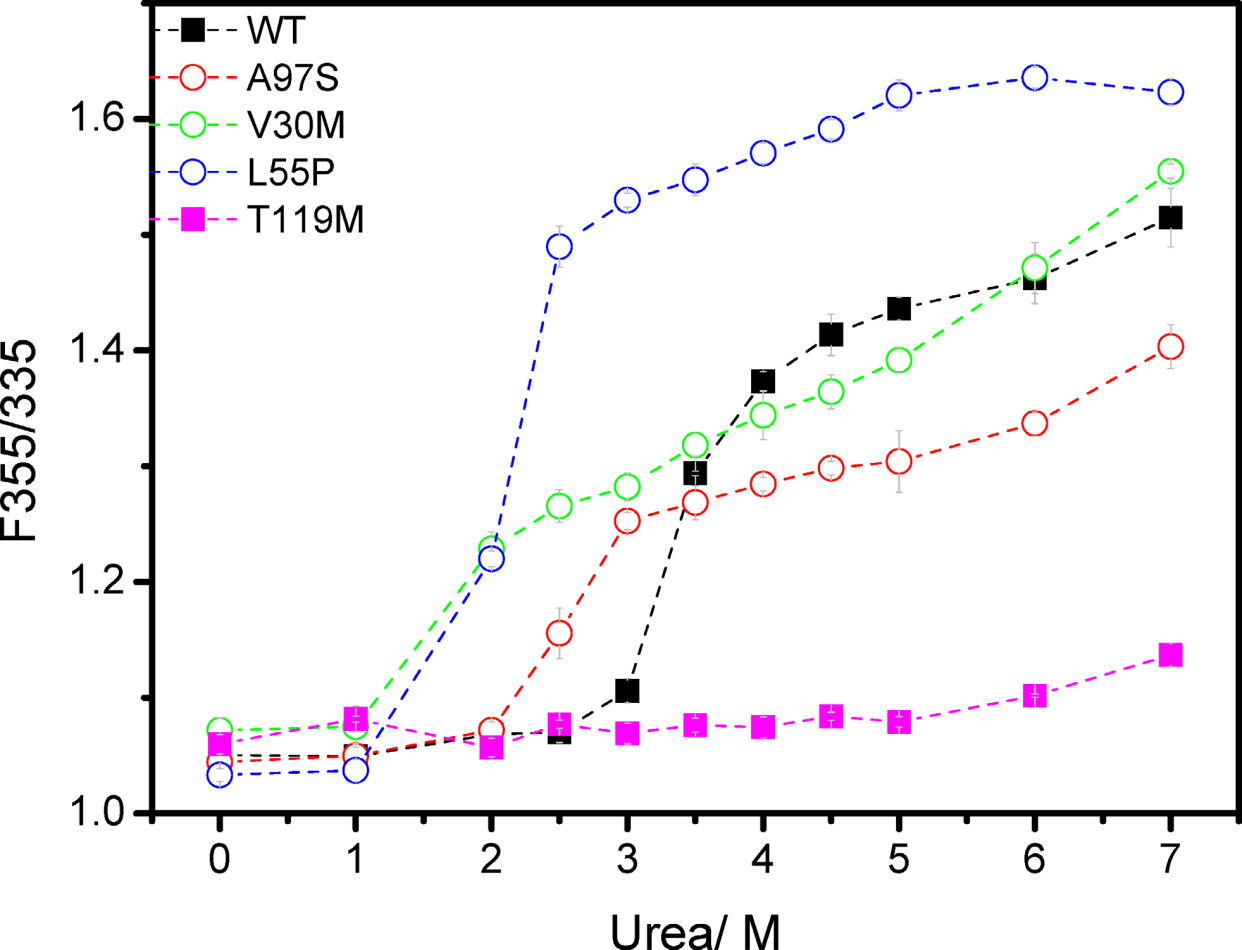
Urea denaturation monitored by tryptophan fluorescence (F355/F335). WT, wild type TTR, black filled squares; A97S‐TTR, red open circles; V30M TTR, green open circles; L55P TTR, blue open circles; T119M TTR, pink filled squares. Dashed lines are drawn to guide the eye.

By monitoring urea denaturation using F355/F335,^21^ we compared the stability of A97S‐TTR with WT‐TTR. Two pathogenic mutants V30M‐TTR and L55P‐TTR, as well as one clinically protective mutant T119M,^11^ were also assessed. V30M‐TTR is the most prevalent disease‐causing variant worldwide^2^, ^3^ and L55P‐TTR is well recognized for its high amyloidogenic potential.^24^, ^25^, ^26^ The dissociation tendency revealed that the highest unfolding change in the L55P‐TTR tetramer, compatible with its notorious amyloidogenic potential (blue open circles in Fig. 1). On the other hand, T119M‐TTR possesses the greatest the tetramer stability against urea‐induced unfolding (pink filled squares in Fig. 1), consistent with the clinical observation of T119M as a protective mutant.^27^, ^28^ V30M‐TTR bears a tetramer stability slightly worse than WT‐TTR (green open circles in Fig. 1), which probably reflects the early‐onset but insidiously progressive disease course. For A97S‐TTR (red open circles in Fig. 1), the result indicates that the tetramer stability of A97S‐TTR is worse than WT‐TTR, confirming the pathogenicity of the mutant. Compared with V30M‐TTR and L55P‐TTR mutants, A97S‐TTR is shown to have slightly better resistivity against urea denaturation.

### Destabilization effect of A97S‐TTR mutant in thermal denaturation

The denaturing temperatures (*Tm*°C) of WT‐TTR and several mutant proteins were measured by DSC. In addition to the three pathogenic mutants A97S‐, V30M‐, and L55P‐ TTR, the proteins carrying R104H and T119M were also studied. The two variants are believed to be protective because TTR M30/H104 and TTR M30/M119 compound heterozygotes can induce structural alterations that increase the stability of the tetramer and protect from amyloid fibril formation.^27^, ^28^ The results are listed in Table 1. In both the conditions of PBS buffer only and PBS buffer containing 5% DMSO, the three amyloidogenic mutants had lower *Tm* than WT‐TTR while the *Tm* of two protective variants were similar to WT‐TTR. Shnyrov VL *et al.* clearly showed the tetrameric forms of non‐amyloidogenic variants had a slightly higher conformational stability than amyloidogenic variants, even though the stability among the amyloidogenic variants did not correlate with the amyloidogenic potential of these proteins.^29^ Our DSC data showed that the stability of A97S‐TTR falls below WT‐TTR and the two protective mutants R104H and T119M. In parallel with the results of chemical denaturation, this finding suggests that A97S‐TTR is an amyloidogenic mutant since it has slightly lower conformational stability. By comparing the *Tm* of TTR proteins measured with and without the presence of tafamidis, we observed an increase in *Tm* of all TTR variants upon tafamidis binding. This observation could serve as evidence of a direct interaction between tafamidis and TTR proteins. Among the three pathogenic mutants, only the *Tm* of A97S‐TTR reached the levels of WT‐TTR and the two protective R104H and T119M variants, implying tafamidis could help A97S‐TTR resume the stability.

**Table 1:** The denaturing temperatures (*Tm* °C) of TTR proteins measured with differential scanning calorimetry (DSC) (60°C/h).

TTR protein	Buffer condition		
	PBS	PBS containing 5% DMSO	PBS containing 5% DMSO and tafamidis
WT‐TTR	101.1 ± 0.3	97.2 ± 0.1	103.8 ± 0.5
V30M‐TTR	92.8 ± 0.1	89.2 ± 0.1	94.8 ± 0.1
L55P‐TTR	97.0 ± 0.2	92.3 ± 0.1	97.0 ± 0.1
A97S‐TTR	98.5 ± 0.1	93.9 ± 0.3	100.0 ± 0.3
R104H‐TTR	101.3 ± 0.1	97.6 ± 0.1	103.5 ± 0.1
T119M‐TTR	102.3 ± 0.1	98.2 ± 0.1	102.2 ± 0.2

### Binding affinity of tafamidis with A97S‐TTR mutant

We then assessed the binding constants of tafamidis to WT‐TTR, A97S‐TTR, V30M‐TTR and L55P‐TTR by ITC. The integrated heat release (kcal/mol) vs. molar ratio of tafamidis added to TTR proteins are listed in Table S2, while the experimental and fitted binding thermograms of ITC experiments are shown in Figure S2. The binding constants of tafamidis to various TTR mutants are listed in Table 2. The binding constants that were obtained consistent with the values previously reported by Bulawa *et al*.^14^ Our results revealed that the binding affinity of tafamidis with A97S‐TTR is comparable to that with WT‐TTR. In contrast, the binding affinities of tafamidis with V30M‐TTR and with L55P‐TTR are significantly weaker.

**Table 2:** The characteristics of isothermal titration calorimetry (ITC) experiments for tafamidis binding to different TTR mutants.

TTR protein	K_a1_ (M^−1^)	ΔH_1_ (kcal/mol)	K_a2_ (M^−1^)	ΔH_2_ (kcal/mol)	K_d1_ (nmol/L)	K_d2_ (nmol/L)
WT	4.2 × 10^8^	−6.48	3.0 × 10^6^	−6.8	2.4	333.3
A97S	3.5 × 10^8^	−5.95	3.8 × 10^6^	−6.6	2.9	263.2
V30M	6.5 × 10^6^	−7.2	1.6 × 10^6^	−6.4	153.8	625
L55P	9 × 10^6^	−8.584	1.2 × 10^6^	−5.12	111	833

The binding isotherm were fitted with a model of two interacting sites exhibiting negative cooperativity to obtain the binding parameters, including dissociation constants, K_d1_ and K_d2_, and enthalpy, ΔH_1_ and ΔH_2_. Dissociation constants of tafamidis:WT‐TTR, tafamidis:A97S‐TTR, tafamidis:V30M‐TTR and tafamidis:L55P‐TTR were determined by finding the best fitted parameters to the experimental thermograms. The binding constants of tafamidis binding K_d1_ and K_d2_ were determined as the inverse of the association constants. All ITC experiments were performed at least three times in order to obtain the statistical results.

### NMR spectroscopic characterization of tafamidis binding to A97S‐TTR mutant

**Figure 2:**
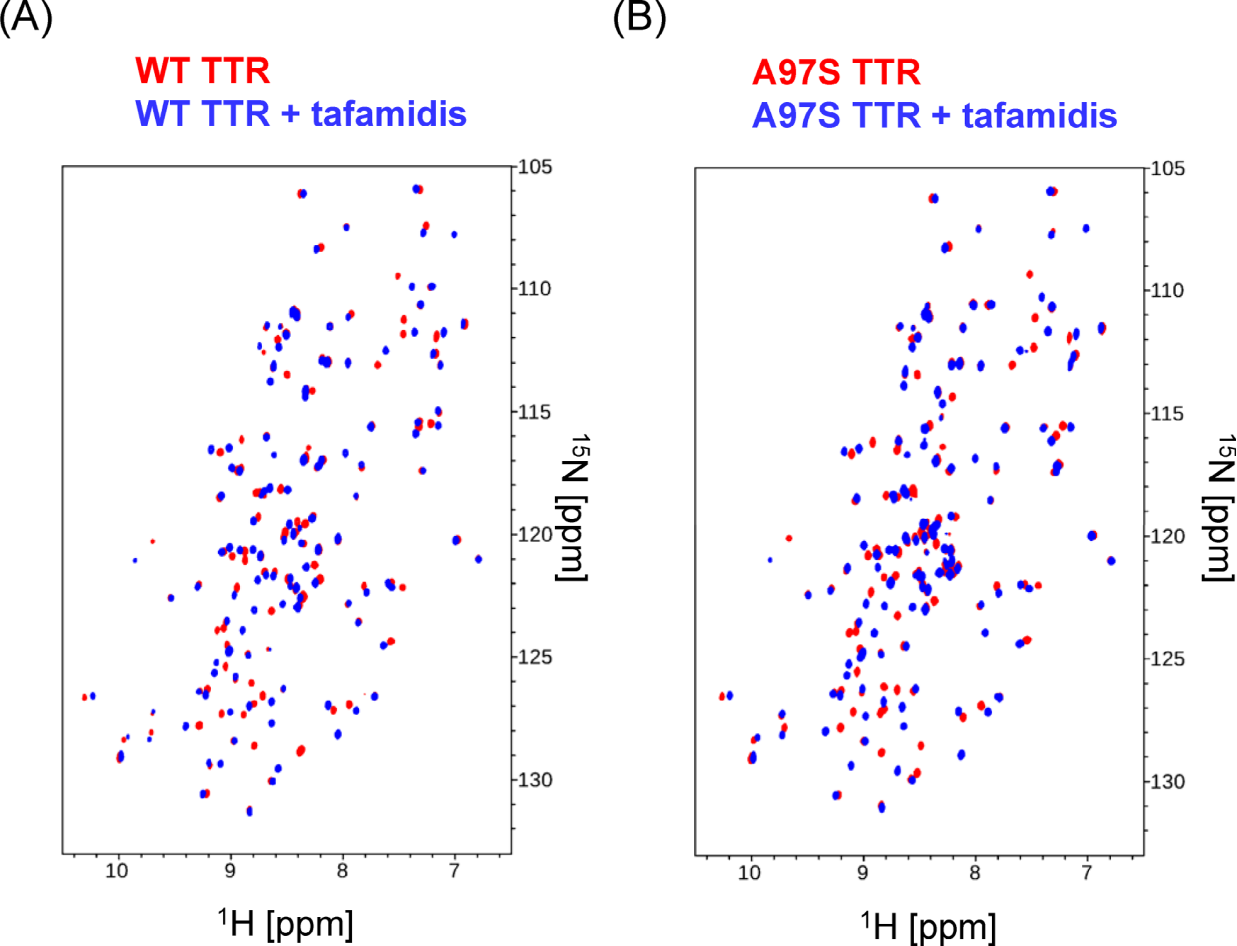
NMR spectroscopic characterization of Tafamidis binding to wildtype transthyretin and A97S transthyretin. (A) 2D TROSY HSQC spectra of ^15^N labeling WT‐TTR with (blue) and without (red) the presence of tafamidis at 2.5:1 molar ratio (tafamidis to protein). (B) 2D TROSY HSQC spectra of ^15^N labeling A97S‐TTR with (blue) and without (red) the presence of tafamidis at 2.5:1 molar ratio (tafamidis to protein).

**Figure 3:**
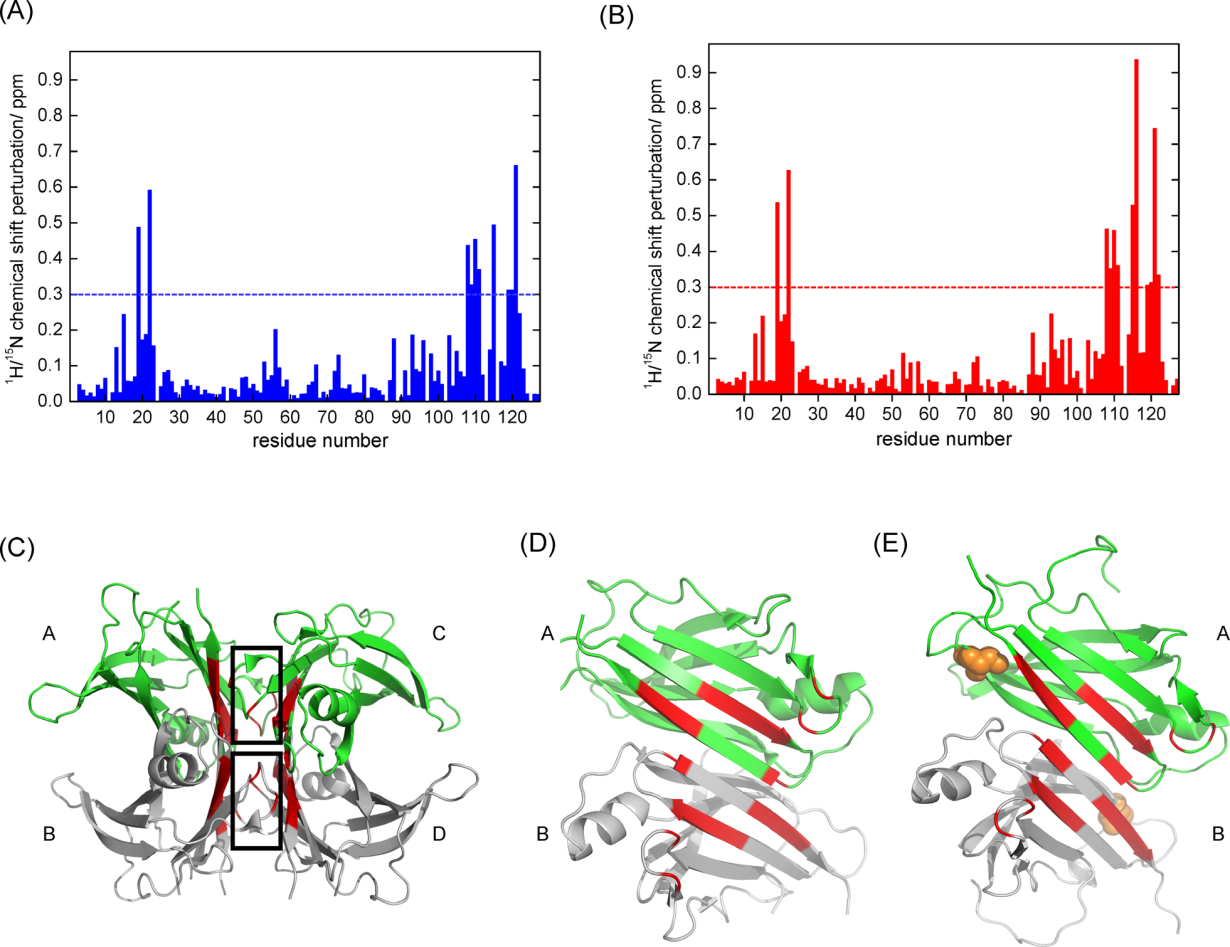
Characterizations of tafamidis binding sites of wildtype transthyretin and A97S transthyretin. (A) Normalized chemical shift changes for each residue of wildtype TTR due to tafamidis binding; (B) normalized chemical shift changes for each residue of A97S‐TTR due to tafamidis binding; (C) mapping of significantly perturbed (>0.3 ppm) residues in red color on the ribbon representation of the wildtype transthyretin using crystal structure (PBD ID: 4pvl), where the thyroxine binding sites are indicated with black box; (D) mapping of significantly perturbed (>0.3 ppm) residues on the ribbon representation of the dimer‐dimer interface of the wildtype transthyretin using crystal structure (PBD ID: 4pvl); (E) mapping of significantly perturbed (>0.3 ppm) residues on the ribbon representation of the dimer‐dimer interface of A97S transthyretin using a homology model generated based on the wildtype transthyretin crystal structure (PBD ID: 4pvl), where Ser‐97 residues are highlighted as orange spheres. In Figure 3D and E, the structural models were turned 90 degrees, with subunits C and B removed, to clearly show the TTR dimer‐dimer interface of subunit A and B.

The ^1^H‐^15^N TROSY‐HSQC spectra of both WT‐TTR and A97S‐TTR samples, with and without the addition of tafamidis, are shown in Figure 2A and B. The resonance peaks showed in the TROSY HSQC spectra are associated mainly with backbone amide groups. The chemical shift perturbation (CSP) of tafamidis binding, to both WT‐TTR and A97S‐TTR are plotted in Figure 3A and B. Those resonance peaks significant shifted as a result of drug binding are mainly located in the dimer‐dimer interface, previously reported as the thyroxine‐binding site by X‐ray crystallography studies.^30^ Thus, the tafamidis binding site of WT‐TTR is well associated with NMR CSP mapping results. Based on the CSP mapping results, tafamidis also affects the chemical shifts of the residues around thyroxine‐binding pocket of A97S‐TTR. To visualize the NMR results, we mapped the significantly perturbed residues on the cartoon presentation of WT‐TTR proteins structure generated with Pymol based on the X‐ray crystal structure (PDB ID: 4pvl)^31^, as shown in Figure 3C.

The four TTR monomer subunits are labeled with A, B, C, and D and the thyroxine binding pocket highlighted with black box. The red‐color highlighted residues are associated with the resonance peaks significantly perturbed upon tafamidis binding. The cartoon presentation of A97S‐TTR structure was generated with Pymol using the homology model created with modeller 9.2 based on the X‐ray crystal structure (PDB ID: 4pvl). By comparing CSP mapping of tafamidis to both WT‐TTR and A97S‐TTR, we show that tafamidis binds to thyroxine binding sites of A97S TTR as well. Together with our ITC results, we concluded that tafamidis also binds strongly and specifically to the pathogenic mutant A97S‐TTR. The chemical shifts of backbone proton, backbone nitrogen and alpha carbon of both WT‐TTR and A97S‐TTR have been documented and uploaded to Biological Magnetic Resonance Data Bank (BMRB). The BMRB entry assigned accession numbers are 27575 and 27576 for WT‐TTR and A97S‐TTR, respectively.

## Discussion

Over the past decade, the advancement of genetic diagnosis has greatly expanded our knowledge of *TTR*‐FAP and facilitated the development of mechanism‐driven therapies targeting to different stages of TTR amyloid formation and deposition, including liver transplantation, TTR stabilizers, gene modifiers like silencing RNA and antisense oligonucleotide therapies.^32^, ^33^, ^34^ Among them, disease‐modifying pharmacotherapy by the TTR stabilizer tafamidis has been proved to be able to significantly slow down the progression of polyneuropathy and deterioration of life quality with a long‐term safety and efficacy.^13^, ^14^, ^15^, ^16^, ^17^, ^18^ Very recently, a multicenter, international, double‐blind phase 3 trial further revealed that tafamidis was associated with reductions in all‐cause mortality and cardiovascular‐related hospitalizations in patients with TTR amyloid cardiomyopathy (TAC).^19^, ^20^

All previous clinical trials were targeted to the patient at the early stage and V30M *TTR*‐FAP was the main recruited genotype. Nevertheless, numerous *TTR* variants other than V30M have been identified and associated with highly variable age of onset, severity and progression rates of the neuropathy and cardiopathy. The wide range of clinical manifestations reflects the diverse pathological effects caused by different *TTR* variants, which further raises the issue that the best treatment may vary with the genotypes. One of the key factors in determining the pathogenicity of a *TTR* variant is its destabilization effect on the tetramer which in turn accelerates amyloid fibril formation. For example, the highly amyloidogenic L55P variant is associated with early‐onset aggressive diffuse amyloidosis and severe cardiac and neurologic dysfunctions, whereas the compound heterozygous carriers with V30M mutation and the protective T119M variant present by a late‐onset and mild phenotype.^35^ This implies that the tetramer stability would influence the treatment response to TTR stabilizers and bring the clinical importance to elucidate the structural changes and related pathogenesis of specific *TTR* variants.

A97S is the predominant genotype of Taiwanese *TTR*‐FAP patients, presenting by a unique phenotype composed of a late‐onset but rapid‐progressive neuropathy and life‐threatening cardiopathy. Nevertheless, there is little information of the impacts of the mutation on the TTR tetramer stabilization and the amyloidogenic potential of the mutant protein. To fill the knowledge gap, we assessed A97S‐TTR tetramer stability against external stresses using both chemical denaturation assay and thermal denaturation assays and compared with the stability of WT‐TTR. Our results revealed that A97S mutation did cause destabilization of the TTR tetramer as the mutated proteins had a higher dissociation tendency and a lower denaturing temperature. Of note, the stability of the late‐onset A97S‐TTR tetramer is still better than that of the V30M‐TTR and L55P‐TTR mutants, both are clinically associated with an early‐onset polyneuropathy. These findings supported that the propensity of destabilization of the TTR tetramer could be one of the determinants of the onset and progression of the disease, indirectly explaining the wide clinical spectrum of *TTR*‐FAP.

In the DSC assay, adding of tafamidis was found to increase the denaturing temperatures of the three pathogenic mutants, indicating that the drug is able to enhance the structural stability. Particularly for the A97S‐TTR, the *Tm* raised up to the level very close to those of WT‐TTR and the protective R104H and T119M variants. The strong affinity between tafamidis and A97S‐TTR was also confirmed by the ITC experiment. As listed in Table 2, our data provided strong evidence that tafamidis can bind to A97S‐TTR with the affinity comparable to WT‐TTR and the binding is much stronger than V30M‐TTR and L55P‐TTR. Together with the DSC result, our ITC result suggested that tafamidis can effectively bind to A97S‐TTR tetramer to provide stabilization effect.

Considering the stability of TTR tetramers against denaturing agents may not reflect their physiologically relevant condition, we then used solution NMR to characterize the structural changes associated with the A97S mutation and the binding effects of tafamidis. By plotting the chemical shift pertubation of tafamidis binding, we documented that tafamidis binds to thyroxine‐binding pocket of A97S‐TTR tetramer, like it behaves to WT‐TTR (Fig. 3). NMR measurements can reveal the spatial and dynamic alternations of the tetramer structures of different pathogenic mutations which are not readily observed in the crystal structures. Solution NMR revealed that AB loop regions interacting with strand A in the DAGH β‐sheet undergo conformational changes, leading to the destabilized β‐sheet.^36^ And the DA β‐structure is believed to be the main site undergoing conformational changes leading to amyloid formation in the pathogenic L55P and V30M mutations.^37^, ^38^ A97 is on the strand G in the DAGH β‐sheet 39 and spatially close to the thyroxine‐binding pocket on the dimer‐dimer interface.^40^ Based on the NMR and ITC experiments, we proposed that the A97S mutation may disrupt β‐sheet structure but the influence can be reversed significantly by tafamidis binding.

To sum up the biophysical characters of A97S‐TTR, destabilization effect of this genetic variant has been clearly observed in both chemical and thermal denaturation. It has been well recognized that the amyloidogenic potential of human pathogenic TTR variants is determined by the destabilization of their native structures. Structural alterations of TTR tetramer induced by genetic variation are essential to induce protein destabilization, the representative early events leading to amyloid fibril dissociations and depositions.^11^ Furthermore, via solution NMR and ITC assessment, this study documented at the conformational destabilization can be reversed though the strong affinity of A97S‐TTR and tafamidis. These findings have several important clinical implications on *TTR*‐FAP. First, our results suggested the therapeutic benefit of tafamidis for the majority of the *TTR*‐FAP patients in Taiwan as A97S is the hot‐spot *TTR* mutation in our population. By providing evidence of the stabilization effect from tafamidis, we hope to raise clinical awareness and to encourage early diagnosis of A97S *TTR*‐FAP. This is also an endeavor in order to strike for more public resources in treatment for this disease. Furthermore, these *in‐vitro* assessments can be applied to address the biophysical features of other rare *TTR* variants. This study provides a feasible approach to evaluate the modulational effects of tafamidis on other rare *TTR* variants.

Although gene modifiers like the RNA interference therapeutic agent and the antisense oligonucleotide are effective in the upstream process of mutant TTR protein production, the safety under a long‐term profound reduction of TTR remains unknown. Although not frequent, two recent large‐scale trials showed severe adverse events under gene modifier therapy could be fatal, including cardiac failure, acute kidney injury and thrombocytopenia.^33^, ^34^ This makes safety of gene modifier therapy a concern for patients with a late onset, rapid progression, or marked cardiac dysfunction, just like the condition of A97S *TTR*‐FAP. On the other hand, a recent meta‐analysis has demonstrated that tafamidis can preserve a better life quality for patients with a rate of adverse events similar to the placebo.^41^ The safety and long‐term efficacy of tafamidis provide the advantage when treating patients of *TTR*‐FAP, therefore, it is important to understand which genetic subtype may benefit more from the drug.

Treatment of *TTR*‐FAP is stepping into a new era as several therapeutic options are now available. Specific genotypes, in association with disease onset and progression, will be important concerns for tailored pharmacotherapy. Our results delineated the biophysical characters of A97S‐TTR tetramer and provided multiple lines of evidence of the modulation effects of tafamidis on its stability. These deepened our understanding of the pathogenesis of A97S *TTR*‐FAP. We hope this study pave a road with stone leading to precision medicine of *TTR* amyloidosis.

## Conflict of Interest

None declared.

## Supporting information


**Figure S1**. Recombinant transthyretin (TTR) expression, purification, and characterization.

**Figure S2**. The experimental and fitted binding thermograms of ITC experiments.

**Table S1** **.** Concentrations of proteins and tafamidis used in isothermal titration calorimetry experiments.
**Table S2** **.** (A) Integrated heat release (kcal/mol) vs. molar ratio of tafamidis added to WT TTR. (B) Integrated heat release (kcal/mol) vs. molar ratio of tafamidis added to A97S TTR. (C) Integrated heat release (kcal/mol) vs. molar ratio of tafamidis added to V30M TTR. (D) Integrated heat release (kcal/mol) vs. molar ratio of tafamidis added to L55P TTR.
