# Supplementary figures and images for: Biophysical characterization and modulation of Transthyretin Ala97Ser
Source: Ann Clin Transl Neurol. 2019 Sep 10;6(10):1961–70. doi: 10.1002/acn3.50887 (PMC6801203; doi:10.1002/acn3.50887)

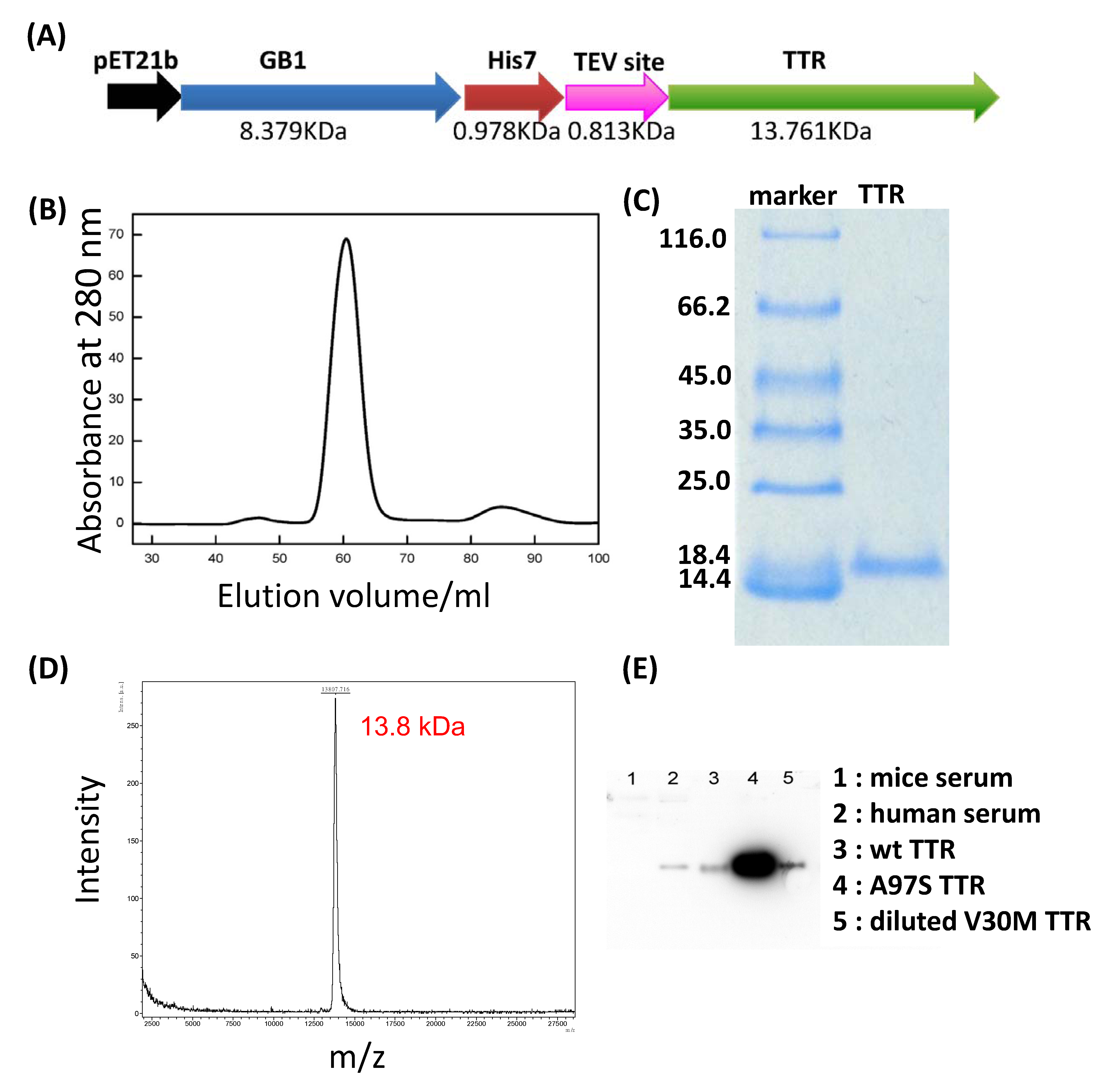

Supplement: Supplementary file 1 — Figure S1. Recombinant transthyretin (TTR) expression, purification, and characterization. [file ACN3-6-1961-s001.tif]

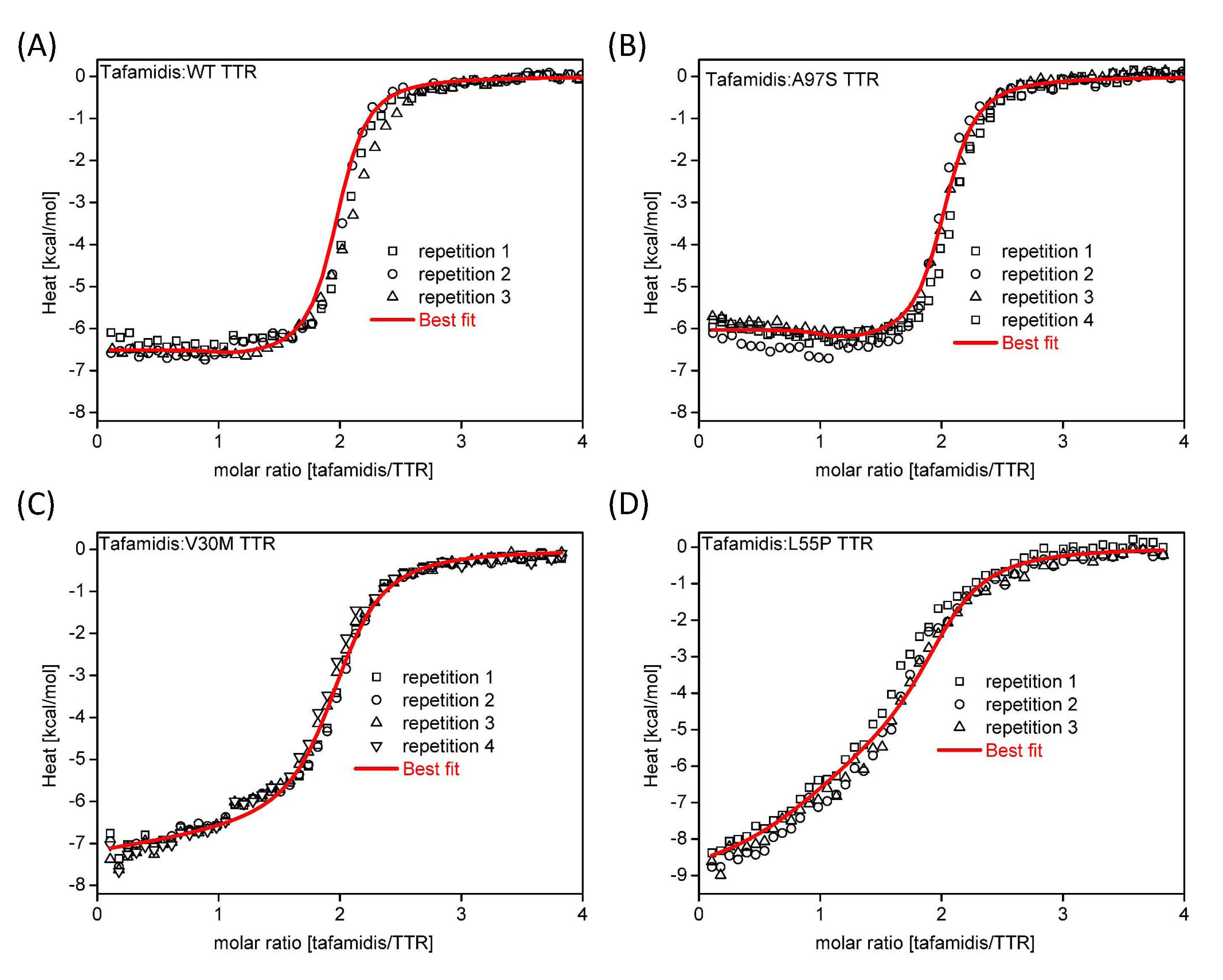

Supplement: Supplementary file 2 — Figure S2. The experimental and fitted binding thermograms of ITC experiments. [file ACN3-6-1961-s002.tif]
