# Supplementary material for: Biophysical characterization and modulation of Transthyretin Ala97Ser
Source: Ann Clin Transl Neurol. 2019 Sep 10;6(10):1961–70. doi: 10.1002/acn3.50887 (PMC6801203; doi:10.1002/acn3.50887)
Supplement: Supplementary file 3 — Table S1 . Concentrations of proteins and tafamidis used in isothermal titration calorimetry experiments. Table S2 . (A) Integrated heat release (kcal/mol) vs. molar ratio of tafamidis added to WT TTR. (B) Integrated heat release (kcal/mol) vs. molar ratio of tafamidis added to A97S TTR. (C) Integrated heat release (kcal/mol) vs. molar ratio of tafamidis added to V30M TTR. (D) Integrated heat release (kcal/mol) vs. molar ratio of tafamidis added to L55P TTR. [file ACN3-6-1961-s003.doc]

**Supplementary figures**

**
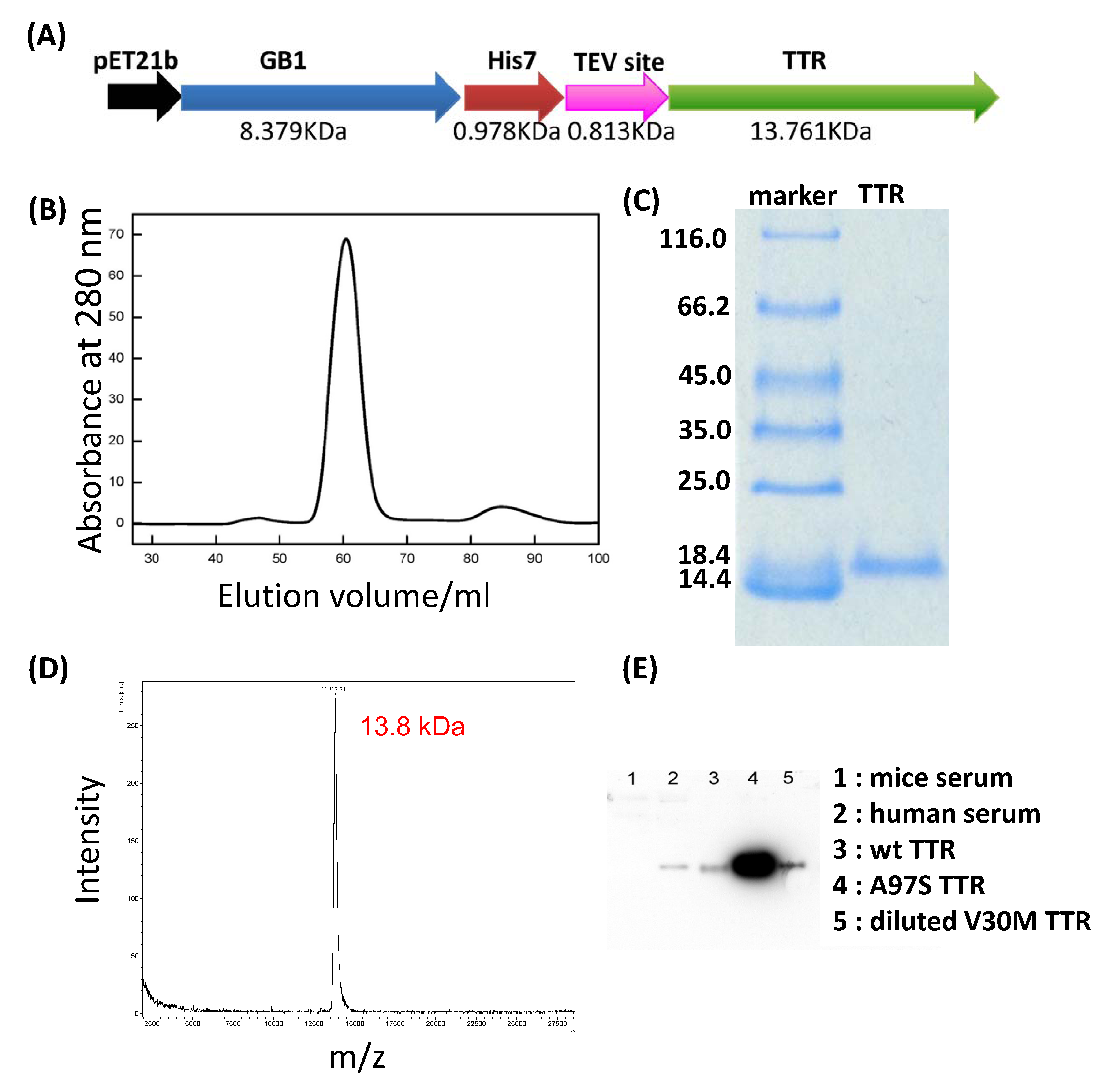
**

**Fig. S1.**

**
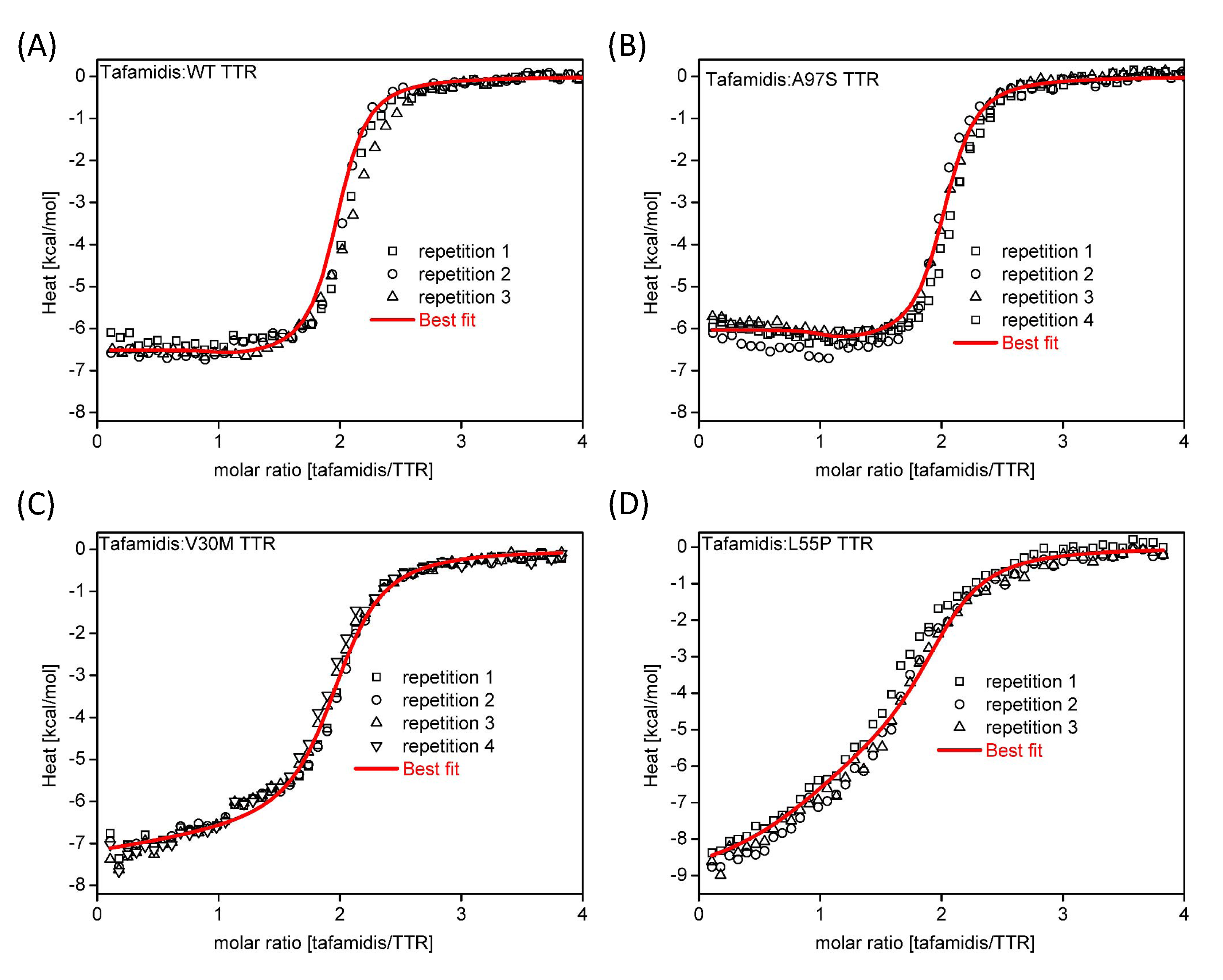
**

**Fig. S2**

**Legends of supplementary figures**

**Fig. S1. Recombinant transthyretin (TTR) expression, purification and characterization.**

The schematic drawing of the design of TTR expression plasmid (A); Gel-filtration profile showing well-resolve elution peak for WT TTR tetramer (B); SDS-PAGE (C), as well as MALDI mass spectrum (D), of purified TTR protein and Western blot showing antibody recognition of recombinant TTR (E).

**Fig. S2. The experimental and fitted binding thermograms of ITC experiments.** Dissociation constants of tafamidis:WT TTR, tafamidis:A97S TTR, tafamidis:V30M TTR and tafamidis:L55P TTR were determined by finding the best fitted parameters to the experimental thermograms.

**Table S1. Concentrations of proteins and tafamidis used in i**sothermal titration calorimetry experiments.

|  | Experiment #1 | Experiment #2 | Experiment #3 | Experiment #4 |
| --- | --- | --- | --- | --- |
| [WT TTR]/ μM | 20.8 | 20.7 | 20.29 |  |
| [Tafamidis]/ μM | 450 | 450 | 450 |  |
| [A97S TTR]/ μM | 20.27 | 21 | 21 |  |
| [Tafamidis]/ μM | 450 | 450 | 450 |  |
| [V30M TTR]/ μM | 15 | 15 | 15 | 15 |
| [Tafamidis]/ μM | 306 | 306 | 306 | 306 |
| [L55P TTR]/ μM | 15 | 15 | 15 |  |
| [Tafamidis]/ μM | 306 | 306 | 306 |  |

**Table S2A. Integrated heat release (kcal/mol) vs. molar ratio of tafamidis added to WT TTR**

| [WT TTR] = 20.8 μM | |  | [WT TTR] = 20.7 μM | |  | [WT TTR] = 20.29 μM | |
| --- | --- | --- | --- | --- | --- | --- | --- |
| Tafamidis :TTR | Heat |  | Tafamidis :TTR | Heat |  | Tafamidis :TTR | Heat |
| 0.113 | -6.095 |  | 0.114 | -6.581 |  | 0.124 | -6.488 |
| 0.189 | -6.211 |  | 0.190 | -6.533 |  | 0.207 | -6.585 |
| 0.266 | -6.101 |  | 0.267 | -6.481 |  | 0.291 | -6.498 |
| 0.342 | -6.408 |  | 0.344 | -6.672 |  | 0.374 | -6.585 |
| 0.419 | -6.334 |  | 0.421 | -6.588 |  | 0.458 | -6.488 |
| 0.496 | -6.279 |  | 0.498 | -6.522 |  | 0.543 | -6.602 |
| 0.573 | -6.436 |  | 0.576 | -6.702 |  | 0.627 | -6.496 |
| 0.651 | -6.292 |  | 0.654 | -6.641 |  | 0.712 | -6.581 |
| 0.729 | -6.417 |  | 0.732 | -6.654 |  | 0.797 | -6.579 |
| 0.807 | -6.361 |  | 0.811 | -6.593 |  | 0.882 | -6.676 |
| 0.885 | -6.504 |  | 0.889 | -6.741 |  | 0.968 | -6.582 |
| 0.964 | -6.374 |  | 0.968 | -6.620 |  | 1.054 | -6.565 |
| 1.043 | -6.436 |  | 1.048 | -6.590 |  | 1.140 | -6.620 |
| 1.122 | -6.161 |  | 1.127 | -6.280 |  | 1.227 | -6.653 |
| 1.201 | -6.305 |  | 1.207 | -6.317 |  | 1.314 | -6.588 |
| 1.281 | -6.231 |  | 1.287 | -6.241 |  | 1.401 | -6.466 |
| 1.361 | -6.220 |  | 1.367 | -6.334 |  | 1.488 | -6.369 |
| 1.441 | -6.171 |  | 1.448 | -6.110 |  | 1.576 | -6.212 |
| 1.521 | -6.204 |  | 1.529 | -6.121 |  | 1.664 | -5.923 |
| 1.602 | -6.201 |  | 1.610 | -6.233 |  | 1.752 | -5.731 |
| 1.683 | -5.934 |  | 1.691 | -5.990 |  | 1.841 | -5.272 |
| 1.764 | -5.902 |  | 1.773 | -5.875 |  | 1.930 | -4.741 |
| 1.846 | -5.524 |  | 1.855 | -5.427 |  | 2.019 | -4.123 |
| 1.928 | -5.056 |  | 1.937 | -4.709 |  | 2.108 | -3.301 |
| 2.010 | -4.017 |  | 2.019 | -3.494 |  | 2.198 | -2.342 |
| 2.092 | -2.854 |  | 2.102 | -2.119 |  | 2.288 | -1.688 |
| 2.175 | -1.822 |  | 2.185 | -1.333 |  | 2.378 | -1.182 |
| 2.257 | -1.169 |  | 2.268 | -0.728 |  | 2.469 | -0.884 |
| 2.340 | -0.938 |  | 2.352 | -0.719 |  | 2.560 | -0.604 |
| 2.424 | -0.554 |  | 2.436 | -0.363 |  | 2.651 | -0.344 |
| 2.507 | -0.424 |  | 2.520 | -0.261 |  | 2.742 | -0.239 |
| 2.591 | -0.518 |  | 2.604 | -0.341 |  | 2.834 | -0.323 |
| 2.675 | -0.363 |  | 2.688 | -0.309 |  | 2.926 | -0.265 |
| 2.760 | -0.287 |  | 2.773 | -0.160 |  | 3.019 | -0.133 |
| 2.845 | -0.314 |  | 2.858 | -0.238 |  | 3.111 | -0.109 |
| 2.929 | -0.259 |  | 2.944 | -0.135 |  | 3.204 | -0.204 |
| 3.015 | -0.139 |  | 3.029 | -0.171 |  | 3.297 | -0.151 |
| 3.100 | -0.070 |  | 3.115 | -0.102 |  | 3.391 | -0.066 |
| 3.186 | -0.193 |  | 3.201 | -0.128 |  | 3.484 | -0.059 |
| 3.272 | -0.118 |  | 3.288 | -0.074 |  | 3.579 | -0.006 |
| 3.358 | -0.066 |  | 3.374 | -0.106 |  | 3.673 | 0.026 |
| 3.445 | -0.014 |  | 3.461 | -0.082 |  | 3.767 | -0.033 |
| 3.531 | 0.022 |  | 3.548 | 0.094 |  | 3.862 | 0.026 |
| 3.618 | 0.019 |  | 3.636 | 0.041 |  | 3.958 | -0.050 |
| 3.706 | 0.044 |  | 3.724 | 0.091 |  | 4.053 |  |
| 3.793 | -0.033 |  | 3.812 | -0.067 |  | 4.149 |  |
| 3.881 | 0.052 |  | 3.900 | 0.055 |  | 4.245 |  |
| 3.969 | -0.061 |  | 3.988 | 0.031 |  | 4.341 |  |
| 4.057 | 0.035 |  | 4.077 | -0.001 |  | 4.438 |  |

Table S2B. Integrated heat release (kcal/mol) vs. molar ratio of tafamidis added to A97S TTR

| [A97S TTR] = 20.27 μM | | |  | [A97S TTR] = 21 μM | | |
| --- | --- | --- | --- | --- | --- | --- |
|  | Exp. #1 | Exp. #2 |  |  | Exp. #1 | Exp. #2 |
| Tafamidis :TTR | Heat | Heat |  | Tafamidis :TTR | Heat #1 | Heat #2 |
| 0.116 | -5.795 | -6.107 |  | 0.112 | -5.712 | -5.967 |
| 0.194 | -5.766 | -6.236 |  | 0.188 | -5.739 | -5.846 |
| 0.273 | -5.889 | -6.161 |  | 0.263 | -5.897 | -5.997 |
| 0.351 | -6.002 | -6.362 |  | 0.339 | -5.892 | -5.950 |
| 0.430 | -5.906 | -6.413 |  | 0.415 | -5.842 | -6.017 |
| 0.509 | -5.928 | -6.422 |  | 0.491 | -5.865 | -6.064 |
| 0.588 | -6.113 | -6.548 |  | 0.568 | -5.954 | -6.145 |
| 0.668 | -6.059 | -6.449 |  | 0.645 | -5.832 | -6.065 |
| 0.748 | -6.147 | -6.462 |  | 0.722 | -5.992 | -6.074 |
| 0.828 | -6.103 | -6.436 |  | 0.799 | -6.069 | -6.193 |
| 0.908 | -6.347 | -6.654 |  | 0.877 | -5.985 | -6.192 |
| 0.989 | -6.159 | -6.690 |  | 0.955 | -6.085 | -6.164 |
| 1.070 | -6.308 | -6.710 |  | 1.033 | -6.063 | -6.214 |
| 1.151 | -6.058 | -6.387 |  | 1.111 | -6.137 | -6.270 |
| 1.232 | -6.129 | -6.458 |  | 1.190 | -6.094 | -6.358 |
| 1.314 | -6.068 | -6.401 |  | 1.269 | -6.198 | -6.307 |
| 1.396 | -6.041 | -6.437 |  | 1.348 | -6.073 | -6.221 |
| 1.478 | -6.149 | -6.335 |  | 1.427 | -6.069 | -6.276 |
| 1.561 | -5.950 | -6.305 |  | 1.507 | -6.041 | -6.213 |
| 1.644 | -6.034 | -6.254 |  | 1.587 | -5.916 | -6.055 |
| 1.727 | -5.861 | -5.939 |  | 1.667 | -5.770 | -5.973 |
| 1.810 | -5.739 | -5.513 |  | 1.748 | -5.512 | -5.744 |
| 1.894 | -5.334 | -4.456 |  | 1.828 | -5.182 | -5.458 |
| 1.978 | -4.694 | -3.383 |  | 1.909 | -4.422 | -4.894 |
| 2.062 | -3.759 | -2.166 |  | 1.991 | -3.667 | -4.097 |
| 2.146 | -2.498 | -1.457 |  | 2.072 | -2.680 | -3.310 |
| 2.231 | -1.668 | -1.048 |  | 2.154 | -2.010 | -2.507 |
| 2.316 | -1.041 | -0.710 |  | 2.236 | -1.337 | -1.722 |
| 2.401 | -0.870 | -0.663 |  | 2.318 | -0.959 | -1.347 |
| 2.487 | -0.563 | -0.384 |  | 2.401 | -0.647 | -0.981 |
| 2.573 | -0.463 | -0.278 |  | 2.484 | -0.408 | -0.581 |
| 2.659 | -0.460 | -0.463 |  | 2.567 | -0.313 | -0.395 |
| 2.745 | -0.277 | -0.296 |  | 2.650 | -0.198 | -0.188 |
| 2.832 | -0.183 | -0.161 |  | 2.734 | -0.136 | -0.189 |
| 2.918 | -0.291 | -0.315 |  | 2.817 | -0.061 | -0.239 |
| 3.006 | -0.198 | -0.065 |  | 2.902 | -0.096 | -0.290 |
| 3.093 | -0.091 | -0.047 |  | 2.986 | -0.089 | -0.058 |
| 3.181 | -0.078 | -0.087 |  | 3.071 | -0.028 | -0.101 |
| 3.269 | -0.173 | -0.240 |  | 3.155 | -0.054 | -0.001 |
| 3.357 | -0.096 | -0.135 |  | 3.241 | 0.019 | -0.048 |
| 3.445 | -0.144 | -0.048 |  | 3.326 | -0.025 | -0.082 |
| 3.534 | -0.025 | -0.100 |  | 3.412 | 0.069 | 0.081 |
| 3.623 | 0.010 | -0.011 |  | 3.498 | 0.068 | 0.042 |
| 3.712 | 0.092 | 0.139 |  | 3.584 | 0.107 | 0.155 |
| 3.802 | 0.023 | 0.058 |  | 3.670 | 0.072 | 0.007 |
| 3.892 | -0.031 | -0.044 |  | 3.757 | 0.066 | 0.076 |
| 3.982 | 0.058 | 0.115 |  | 3.844 | 0.135 | 0.116 |
| 4.072 | 0.003 | 0.011 |  | 3.931 | -0.001 | 0.049 |
| 4.163 | 0.000 | 0.000 |  | 4.019 | 0.161 | -0.015 |

Table S2C. Integrated heat release (kcal/mol) vs. molar ratio of tafamidis added to V30M TTR

| [V30M TTR] = 15 μM | | | | |
| --- | --- | --- | --- | --- |
|  | Exp. #1 | Exp. #2 | Exp. #3 | Exp. #4 |
| Tafamidis :TTR | Heat | Heat | Heat | Heat |
| 0.118 | -6.761 | -6.951 | -7.379 | -7.031 |
| 0.197 | -7.359 | -7.558 | -7.622 | -7.669 |
| 0.276 | -7.030 | -7.086 | -7.320 | -7.257 |
| 0.356 | -7.065 | -7.007 | -7.217 | -7.211 |
| 0.436 | -6.796 | -7.007 | -6.940 | -7.075 |
| 0.516 | -7.090 | -7.055 | -7.260 | -7.154 |
| 0.596 | -6.923 | -6.938 | -7.020 | -7.049 |
| 0.677 | -6.960 | -6.824 | -6.973 | -7.034 |
| 0.758 | -6.623 | -6.590 | -6.740 | -6.749 |
| 0.839 | -6.716 | -6.673 | -6.709 | -6.786 |
| 0.921 | -6.674 | -6.526 | -6.744 | -6.726 |
| 1.002 | -6.640 | -6.584 | -6.668 | -6.711 |
| 1.084 | -6.582 | -6.603 | -6.554 | -6.617 |
| 1.167 | -6.469 | -6.372 | -6.432 | -6.487 |
| 1.249 | -6.076 | -6.061 | -6.013 | -5.992 |
| 1.332 | -6.094 | -6.025 | -6.035 | -6.055 |
| 1.415 | -5.938 | -5.930 | -5.937 | -5.997 |
| 1.499 | -5.903 | -5.812 | -5.859 | -5.842 |
| 1.582 | -5.764 | -5.685 | -5.652 | -5.662 |
| 1.666 | -5.693 | -5.765 | -5.587 | -5.659 |
| 1.750 | -5.544 | -5.608 | -5.509 | -5.402 |
| 1.835 | -5.394 | -5.356 | -5.116 | -4.936 |
| 1.920 | -5.158 | -5.125 | -4.825 | -4.623 |
| 2.005 | -4.652 | -4.704 | -4.149 | -3.888 |
| 2.090 | -4.258 | -4.330 | -3.728 | -3.471 |
| 2.176 | -3.413 | -3.538 | -2.929 | -2.669 |
| 2.262 | -2.630 | -2.844 | -2.387 | -2.112 |
| 2.348 | -1.915 | -2.007 | -1.720 | -1.443 |
| 2.434 | -1.605 | -1.695 | -1.521 | -1.447 |
| 2.521 | -1.178 | -1.206 | -1.259 | -1.154 |
| 2.608 | -0.814 | -0.919 | -0.930 | -0.934 |
| 2.695 | -0.699 | -0.762 | -0.800 | -0.691 |
| 2.782 | -0.553 | -0.657 | -0.615 | -0.573 |
| 2.870 | -0.534 | -0.593 | -0.584 | -0.563 |
| 2.958 | -0.416 | -0.494 | -0.493 | -0.442 |
| 3.047 | -0.385 | -0.434 | -0.502 | -0.368 |
| 3.135 | -0.330 | -0.296 | -0.346 | -0.290 |
| 3.224 | -0.313 | -0.360 | -0.293 | -0.323 |
| 3.313 | -0.349 | -0.302 | -0.341 | -0.406 |
| 3.403 | -0.269 | -0.268 | -0.249 | -0.280 |
| 3.492 | -0.252 | -0.244 | -0.229 | -0.223 |
| 3.582 | -0.177 | -0.333 | -0.197 | -0.271 |
| 3.673 | -0.164 | -0.186 | -0.269 | -0.232 |
| 3.763 | -0.200 | -0.173 | -0.077 | -0.166 |
| 3.854 | -0.247 | -0.223 | -0.228 | -0.116 |
| 3.945 | -0.260 | -0.228 | -0.318 | -0.335 |
| 4.036 | -0.071 | -0.128 | -0.163 | -0.089 |
| 4.128 | -0.172 | -0.205 | -0.242 | -0.230 |
| 4.220 | -0.211 | -0.174 | -0.078 | -0.100 |

**Table S2D. Integrated heat release (kcal/mol) vs. molar ratio of tafamidis added to L55P TTR**

| [L55P TTR] = 15 μM | | | |
| --- | --- | --- | --- |
|  | Exp. #1 | Exp. #2 | Exp. #3 |
| Tafamidis :TTR | Heat | Heat | Heat |
| 0.107 | -8.379 | -8.761 | -8.613 |
| 0.179 | -8.320 | -8.765 | -8.989 |
| 0.251 | -8.061 | -8.453 | -8.208 |
| 0.323 | -8.002 | -8.555 | -8.374 |
| 0.395 | -7.926 | -8.370 | -8.219 |
| 0.468 | -7.643 | -8.425 | -8.142 |
| 0.541 | -7.717 | -8.320 | -8.064 |
| 0.614 | -7.488 | -7.947 | -7.722 |
| 0.687 | -7.342 | -7.834 | -7.450 |
| 0.761 | -7.233 | -7.713 | -7.501 |
| 0.835 | -6.904 | -7.420 | -7.206 |
| 0.909 | -6.581 | -6.860 | -7.030 |
| 0.983 | -6.385 | -7.122 | -6.928 |
| 1.058 | -6.365 | -6.958 | -6.613 |
| 1.133 | -6.269 | -6.778 | -6.826 |
| 1.208 | -5.817 | -6.503 | -6.324 |
| 1.283 | -5.482 | -6.057 | -5.825 |
| 1.359 | -5.414 | -6.135 | -6.088 |
| 1.435 | -4.848 | -5.706 | -5.523 |
| 1.511 | -4.552 | -5.070 | -5.475 |
| 1.587 | -4.031 | -4.999 | -4.760 |
| 1.664 | -3.244 | -4.088 | -4.218 |
| 1.741 | -2.933 | -3.492 | -3.715 |
| 1.818 | -2.449 | -3.085 | -3.175 |
| 1.895 | -2.191 | -2.311 | -2.768 |
| 1.973 | -1.683 | -2.215 | -2.372 |
| 2.050 | -1.587 | -2.039 | -2.069 |
| 2.129 | -1.344 | -1.676 | -1.798 |
| 2.207 | -1.181 | -1.345 | -1.464 |
| 2.285 | -0.964 | -1.228 | -1.232 |
| 2.364 | -0.770 | -1.077 | -1.207 |
| 2.443 | -0.714 | -0.872 | -0.839 |
| 2.523 | -0.668 | -1.029 | -0.959 |
| 2.602 | -0.442 | -0.733 | -0.751 |
| 2.682 | -0.293 | -0.586 | -0.833 |
| 2.762 | -0.189 | -0.455 | -0.410 |
| 2.843 | -0.028 | -0.333 | -0.509 |
| 2.923 | -0.229 | -0.516 | -0.499 |
| 3.004 | -0.048 | -0.375 | -0.196 |
| 3.085 | 0.014 | -0.120 | -0.279 |
| 3.166 | -0.103 | -0.326 | -0.274 |
| 3.248 | -0.025 | -0.241 | -0.404 |
| 3.330 | 0.069 | -0.241 | -0.211 |
| 3.412 | 0.016 | -0.266 | -0.275 |
| 3.494 | -0.017 | -0.188 | -0.190 |
| 3.577 | 0.217 | -0.027 | -0.070 |
| 3.660 | 0.140 | -0.063 | -0.067 |
| 3.743 | 0.136 | -0.249 | -0.144 |
| 3.826 | -0.001 | -0.220 | -0.217 |

**Supplementary methods**

***hTTR* gene sequence**

The human transthyretin (*TTR*) gene was codon optimized and synthesized by Genomics BioSci & Tech (New Taipei City). The DNA sequence of synthetic *hTTR* gene is listed below.

GGTCCGACGGGTACGGGTGAAAGCAAATGTCCGCTGATGGTCAAAGTTCTGGATGCAGTTCGTGGTTCTCCGGCAATCAATGTGGCTGTCCATGTATTTCGTAAAGCGGCCGATGACACTTGGGAACCGTTCGCGAGCGGTAAAACAAGCGAGTCTGGCGAACTGCACGGTCTGACCACGGAAGAAGAATTTGTTGAGGGGATCTACAAAGTGGAAATCGATACCAAAAGTTACTGGAAAGCACTGGGCATTTCCCCGTTTCATGAGCACGCAGAAGTGGTTTTCACCGCTAACGACAGCGGGCCACGTCGCTATACGATCGCCGCACTGCTGAGCCCGTATTCCTACTCCACCACCGCCGTTGTCACGAACCCGAAAGAATAA

**TTR protein expression construct**

The synthetic *hTTR* gene was cloned by restriction-free cloning method into pET21b expression vector, containing GB1 tag, followed by 7x Histidine tag and the cleavage site of tobacco etch virus (TEV) protease, at the N-terminal. The final expression vector carries GB1-His-His-His-His-His-His-His-TEV-TTR, as shown in Figure S1a. Standard site-directed mutagenesis experiments were performed to obtain the expression constructs of different TTR mutants.

**TTR protein production, purification and characterizations.**

All TTR proteins, including WT-TTR and other variants, were expressed in *Escherichia coli* BL21(DE3) competent cells by induction with 0.5 mM IPTG (Gold Biotechnology) at 37 ^o^C for 5-6 hours. Cells were resuspended in lysis buffer (50 mM Na phosphate, 300 mM NaCl, 10 mM imidazole, and 7 mM β-mercaptoethanol, pH 8.0) and lysed by sonication. Supernatant obtained after centrifugation at 15,500 rpm (JA 25.50 rotor, Beckman) for 45 minutes at 4°C was purified using nickel affinity column, pre-equilibrated with the equilibration buffer (50 mM Na phosphate, 300 mM NaCl, 10 mM imidazole, and 7 mM β-mercaptoethanol, pH 8.0). The resin was washed with series of wash buffers (50 mM Na phosphate, 300 mM NaCl, 10 mM imidazole, and 7 mM β-mercaptoethanol, pH 8.0; 50 mM Na phosphate, 300 mM NaCl, 20 mM imidazole, and 7 mM β-mercaptoethanol, pH 8.0). Protein was finally eluted with buffer containing 50 mM Na phosphate, 300 mM NaCl, 250 mM imidazole, and 7 mM β-mercaptoethanol, pH 8.0. Protein was then exchanged to buffer containing 20 mM Na phosphate, 150 mM NaCl, 10% glycerol and then stored at −80°C. To obtain tetrameric TTR protein without his-tag, TEV protease was added to fused protein (GB1-TTR:TEV=5:1) at 30 °C and incubated for 40-45hr before further purification using size exclusion chromatography (Superdex 200, GE Healthcare). Using wild-type TTR as an example, well-resolved elution peak containing tetrameric TTR protein was obtained as shown in Fig. S1(b). The characterizations using SDS-PAGE, MALDI Mass spectrometery and Western blot analysis, shown in Fig. S1(c), Fig. S1(d) and Fig. S1(e), confirmed that correct protein was obtained.

**Isothermal titration calorimetry (ITC)**

Isothermal titration calorimetry experiments were performed according to Bulawa *et al* with modification^14^. Dissociation constants for tafamidis:WT-TTR, tafamidis:A97S TTR, tafamidis:V30M TTR and tafamidis:L55P TTR were determined using a Microcal VP-ITC isothermal titration calorimeter (Microcal Inc.). Tafamidis was dissolved in ITC buffer (100 mM Na phosphate, 100 mM KCl, 1 mM EDTA, 5% DMSO, pH 7.6) and titrated into an ITC cell containing TTR protein in the same buffer. The concentrations of tafamidis and various TTR proteins for ITC experiments are documented in Table S1. The initial injection of 2.5 µL of tafamidis solution was followed by 49 injections of 5 µL each (25°C), and the heat evolved was recorded as a function of time after each injection. The blank experiments were carried out exactly the same way except that no TTR protein was included in ITC cell. After subtracting the blank, the total heat released for each injection was obtained by integrating each injection peak. Tables S2A to S2D document the experimental thermograms, the total heat release of each injection versus the molar ratio of tafamidis added to TTR in ITC cell. The experimental and fitted thermograms were plotted in Fig. S1.
