# Supplementary material for: Biophysical characterization and modulation of Transthyretin Ala97Ser
Source: Ann Clin Transl Neurol. 2019 Sep 10;6(10):1961–70. doi: 10.1002/acn3.50887 (PMC6801203; doi:10.1002/acn3.50887)
Supplement: Supplementary file 4 [file ACN3-6-1961-s004.docx]

Fig. S1. Recombinant transthyretin (TTR) expression, purification and characterization.

The schematic drawing of the design of TTR expression plasmid (A); Gel-filtration profile showing wellresolve

elution peak for WT TTR tetramer (B); SDS-PAGE (C), as well as MALDI mass spectrum (D), of

purified TTR protein and Western blot showing antibody recognition of recombinant TTR (E).

Fig. S2. The experimental and fitted binding thermograms of ITC experiments. Dissociation constants of

tafamidis:WT TTR, tafamidis:A97S TTR, tafamidis:V30M TTR and tafamidis:L55P TTR were determined by

finding the best fitted parameters to the experimental thermograms.
